# Practical Notes and Formulæ

**Published:** 1879-12-20

**Authors:** 


### PRACTICAL NOTES AND FORMULÆ.

**Dysmenorrhœa.**—A writer in Medical Brief says:

"My daughter has been accustomed to suffer a little when it was time for her courses to appear, but when it did appear was always very scant, and she would always bleed at the nose."

So we see when the nisus hemorrhagicus should relieve the hyperæmia of the reproductive tissues epistaxis would set in and relieve the system in a way that was not conducive to health. This state of affairs had been going on for several years, until the catamenia and epistaxis failed to show themselves and resulted in the mother sending after me. I ordered turpentine stupes to be used and administered:

℞ Camphor.......................................... 10 grains.
Dover's powders.................................... 10 grains.
Ext. hyoscyamus.................................... 10 grains.
Mix. and ft. pill 10.

Dose, two every two hours.

Now, to relieve her from her condition, I ordered viburnum prunifolium, one drachm three times a day. She is now well and healthy.

**Chronic Rheumatism.**—Dr. Ellingwood, in Brief, says: I give the following as the treatment from which I have obtained the best results in chronic rheumatism:

℞. Macrotin...................40 grains.
Pulv. guaiacum............. 1 drachm.
M. Fill capsules No. XII. Sig.: Take 1 capsule every 4 hours.

In addition to the above, and, in my opinion, the most important of all measures where its use is practicable, pass a strong electric current through the part, for at least an hour, every day or two. I have used the "alkaline course" usually prescribed, and in fact the large proportion of the one hundred and one agents mentioned in the National Dispensatory, as useful in such cases, and have met with the best success with the course prescribed.

**Churchill's Tinct. of Iodine.**—Prof. Parvin recently reports the formula for Churchill's tincture, and which we remember some time since he pronounced the most universally applicable of all topical applications in the treatment of uterine disorders.

℞. Iodine...... ................℥ ijss.

Iodid potass................ʒ ij.

Spt. rectificat..............f. ℥ xii.

Alcohol............... .........℥ iv.

This is the formula as given in Churchill's last edition of his work on Diseases of Women.

**Good Formula for Administering Podophyllin.**—℞. Podophyllin, gr. ij; essential zingiberis, ʒij; spts. vini rectif, ℥ij. Fiat guttæ. A teaspoonful to a wineglass full of water every night or second night, as required.—*Medical Summary.*

**Treatment of Dropsy.**—I would say that, as a diuretic in dropsical cases, generally, the following is, incomparably, the best I have tried during a practice of nearly thirty years:

℞. Potassii bromidi....................................
Potassii acetatis ...............................aa ℥ j
Potassii iodidi....................................
Pulv. potassii chloratis............................
Pulv. potassii bicarbonatis.........................aa ʒ iij
Tr. digitalis purpu..................................f ℥ j
Alcoholis.............................................f ℥ ij
Aquæ..........................................q. s.
Shake to saturation.
Sig.—Coch. mag., ter die.

The medicine should be taken before eating, in water, flaxseed or watermelon seed tea. Give the above combination a trial before resorting to elaterium or paracentesis. Once upon a time, and while indulging in a random search for something with which to "hit the nail on the head," I determined to aggregate the forces of the above array. I was most agreeably disappointed, and in every case since the result has been truly pleasing. In no instance has the effusion refused to disappear or to greatly decrease, promptly and rapidly, on first trial, usually in three or four days, and in one case in twenty-four hours. Under some conditions, a longer time will, of course, be demanded. In two cases, perhaps, where it became necessary to recur for a second or third time to the use of the remedy, the effect was not so gratifying.

In conclusion, I will add, that while I do not give the prescription as curative, per se, in any considerable number of cases, I hesitate not to express my anxiety to have the others test its virtues, believing that it will, as a basilar factor, be found most satisfactorily potential.—Dr. Twitty, in *Med. aud Surg. Reporter.*

**Post Nasal Catarrh.**—Dr. Spicer, in Medical Brief, says: Having had some experience in the above disease, and knowing it to be very rebellious to all remedial agents, I have had better success with the following:

℞. Potassii iodidi...................2 drachms.
Syrup sarsaparilla..............3 ounces.
M. Sig.: Teaspoonful three times per day before each meal.

Have the patient to snuff up the nose twice daily a teaspoonful of the following formula:

℞. Ol. sassafras..........................2 drachms.
Glycerine...............................4 ounces.

**Sycosis.**—In the beginning of sycosis, when there is mainly the deep-seated burning or tingling, with possibly a few scattered postules, and some tenderness when the hairs are seized and pushed in vertically, a cooling treatment may be of service, and quite an amount of relief is obtained by the following lotion, which alone may arrest the disease: ℞.—Liquor. plumbi acet. dil., ʒii; pulv. calamin. prep. zinci oxidi, aa ʒi; glycerin, ʒii; aquæ rosæ, ℥ijss. This is to be well shaken, and the parts kept moistened with it much of the time.

**Pavor Nocturnus, or "Night Terrors,"** says Wertheimer, occur almost exclusively in children of anæmic constitution, and of nervous temperament. The most prominent symptoms—sudden waking in the first few hours of sleep, with loud, anxious cries, partial and momentary loss of consciousness, with a return to sleep after a thorough awakening—he attributes to a temporary increased excitability of the brain. One peculiarity of these cases, says the author, is that the patients have no recollection of the occurrence of these frightful dreams.

After the twelfth year they usually pass away; the prognosis is, therefore, always favorable, even if no medication be resorted to.

The proper therapy consists in increasing the nutritive powers and in the administration of quinia, iron and bromide of potassium.—*St. Petersburg Med. Wochenschrift*, No. 36, 1879.

**Sulphuric Acid in Cholera.**—Dr. MacCormac, of Belfast, who had great experience in cholera in India, has urged on the English government the prophylactic treatment by dilute sulphuric acid—

℞. Acidi sulphurici......................gtt. vj
Aquæ......................................℥j. M.

Sig.—This amount in a cup of mint water once or twice daily.

Both in India and the United States this has proved itself the best known prophylactic.—*Medical and Surgical Reporter.*

**Camphorated Dover's Powder.**—

Powdered opium.........................10 grains.
Powdered Camphor.......................2 scruples.
Powdered ipecac.........................1 scruple.
Powdered cream of tartar............8 scruples.

Mix intimately. Dose, from five to ten grains. The powder contains a little less than half the proportion of opium present in the officinal Dover's powder.—*Druggists' Circular.*

**An Anti-Asthmatic Powder.**—The Practicien (a new French medical weekly) gives the following formula, which has been adopted with much success by Dr. de Creveiser, of Briey. Take equal weights of stramonium, sage, belladonna, and digitalis; crush to about the coarseness of sawdust, damp a little, and mix in as much nitre as of one or the other substances. Burn a little on a plate, cover with a paper cone open at the top, and let the sufferer inhale the smoke. If the smoke is too abundant, damp the mixture with a little water.

**Syphiloderma.**—In chancres, especially about the face, much more rapid removal than otherwise may be obtained by keeping them covered with the emplastrum hydrargyri of the Germans, made thus: ℞.—Hydrarg., ℥i; terebinth. commun., ℥j; ceræ flavæ, ℥jss; emplast. plumbi, ℥vj. Powdered iodoform dusted on yields good results, but I seldom employ it on account of the odor.—*Archives of Dermatology.*

**Treatment of Dropsy.**—I would say that, as a diuretic in dropsical cases, generally, the following is, incomparably, the best I have tried during a practice of nearly thirty years:

℞. Potassii bromidi....................................
Potassii acetatis ..............................aa ℥ j
Potassii iodidi....................................
Pulv. potassii chloratis..............................
Pulv. potassii bicarbonatis.......................aa ʒ iij
Tr. digitalis purpu.................................f ℥ j
Alcoholis.........................................f ℥ ij
Aquæ.......................................q. s.
Shake to saturation.
Sig.—Coch. mag., ter die.

The medicine should be taken before eating, in water, flaxseed or watermelon seed tea. Give the above combination a trial before resorting to elaterium or paracentesis. Once upon a time, and while indulging in a random search for something with which to "hit the nail on the head," I determined to aggregate the forces of the above array. I was most agreeably disappointed, and in every case since the result has been truly pleasing. In no instance has the effusion refused to disappear or to greatly decrease, promptly and rapidly, on first trial, usually in three or four days, and in one case in twenty-four hours. Under some conditions, a longer time will, of course, be demanded. In two cases, perhaps, where it became necessary to recur for a second or third time to the use of the remedy, the effect was not so gratifying.

In conclusion, I will add, that while I do not give the prescription as curative, per se, in any considerable number of cases, I hesitate not to express my anxiety to have the others test its virtues, believing that it will, as a basilar factor, be found most satisfactorily potential.—Dr. Twitty, in *Med. aud Surg. Reporter.*

**Post Nasal Catarrh.**—Dr. Spicer, in Medical Brief, says: Having had some experience in the above disease, and knowing it to be very rebellious to all remedial agents, I have had better success with the following:

℞. Potassii iodidi....................2 drachms.
Syrup sarsaparilla...............3 ounces.
M. Sig.: Teaspoonful three times per day before each meal.

Have the patient to snuff up the nose twice daily a teaspoonful of the following formula:

℞. Ol. sassafras..........................2 drachms.
Glycerine..........................4 ounces.

**Sycosis.**—In the beginning of sycosis, when there is mainly the deep-seated burning or tingling, with possibly a few scattered postules, and some tenderness when the hairs are seized and pushed in vertically, a cooling treatment may be of service, and quite an amount of relief is obtained by the following lotion, which alone may arrest the disease: ℞.—Liquor. plumbi acet. dil., ʒii; pulv. calamin. prep. zinci oxidi, aa ʒi; glycerin, ʒii; aquæ rosæ, ℥ijss. This is to be well shaken, and the parts kept moistened with it much of the time.

**Pavor Nocturnus, or "Night Terrors,"** says Wertheimer, occur almost exclusively in children of anæmic constitution, and of nervous temperament. The most prominent symptoms—sudden waking in the first few hours of sleep, with loud, anxious cries, partial and momentary loss of consciousness, with a return to sleep after a thorough awakening—he attributes to a temporary increased excitability of the brain. One peculiarity of these cases, says the author, is that the patients have no recollection of the occurrence of these frightful dreams.

After the twelfth year they usually pass away; the prognosis is, therefore, always favorable, even if no medication be resorted to.

The proper therapy consists in increasing the nutritive powers and in the administration of quinia, iron and bromide of potassium.—*St. Petersburg Med. Wochenschrift*, No. 36, 1879.

**Sulphuric Acid in Cholera.**—Dr. MacCormac, of Belfast, who had great experience in cholera in India, has urged on the English government the prophylactic treatment by dilute sulphuric acid—

℞. Acidi sulphurici....................gtt. vj
Aquæ....................................℥j. M.

Sig.—This amount in a cup of mint water once or twice daily.

Both in India and the United States this has proved itself the best known prophylactic.—*Medical and Surgical Reporter.*

**Camphorated Dover's Powder.**—

Powdered opium........................10 grains.
Powdered Camphor......................2 scruples.
Powdered ipecac.......................1 scruple.
Powdered cream of tartar..............8 scruples.

Mix intimately. Dose, from five to ten grains. The powder contains a little less than half the proportion of opium present in the officinal Dover's powder.—*Druggists' Circular.*

**An Anti-Asthmatic Powder.**—The Practicien (a new French medical weekly) gives the following formula, which has been adopted with much success by Dr. de Creveiser, of Briey. Take equal weights of stramonium, sage, belladonna, and digitalis; crush to about the coarseness of sawdust, damp a little, and mix in as much nitre as of one or the other substances. Burn a little on a plate, cover with a paper cone open at the top, and let the sufferer inhale the smoke. If the smoke is too abundant, damp the mixture with a little water.

**Syphiloderma.**—In chancres, especially about the face, much more rapid removal than otherwise may be obtained by keeping them covered with the emplastrum hydrargyri of the Germans, made thus: ℞.—Hydrarg., ℥i; terebinth. commun., ℥j; ceræ flavæ, ℥jss; emplast. plumbi, ℥vj. Powdered iodoform dusted on yields good results, but I seldom employ it on account of the odor.—*Archives of Dermatology.*

**Practical Notes.**—Dr. J. S. Jones, of Jackson, La., writes: "I believe Labarruque's solution is a specific for poison oak eruption."

He further says that as a liniment or revulsive for external application, he has found no "pain killer" so hot and so efficient as the following:

℞. Chloral......................½ ounce.

Tinc. capsicum..........2 ounces.

M. Apply over the region of pain. He adds: "It is not *quite* as hot as fire."

For erysipelas, Dr. Jones says: When the quarantine of 1878 prevented us getting cranberries, I substituted for them poultices of the common wild plum for erysipelas, with the ordinary internal treatment, and have found no remedy equal to it. I want nothing better; the only trouble is we can only get the plums during the fall. Tell your readers to try them. Cook with corn meal, and apply hot or cold as the patient likes.

**To Prevent Malarial Fevers.**—Dr. Q. C. Smith, of California, publishes the following as an excellent preventive of chills and fever. Briefly stated, the formula is this:

℞. Powd. Chinchona bark,

Powd. iodide potassium, aa ℥j;

Powd. ipecac, ʒij;

Whisky, one pint.

Mix; and take a teaspoonful three times a day, just after meals. This is the adult dose.

He says it is a preparation, the knowledge of which is certainly well worthy of being perpetuated, as it is certainly very efficacious for the purpose for which it is recommended.

**Yerba Santa in Catarrhal Affections.**—Dr. J. C. Wilson, in Philadelphia Hospital, says: The remedial virtues of the plant are contained in the gum-resin which is abundantly yielded by the leaves.

It is sold in the shops in the form of a fluid extract, the taste of which is aromatic, sweetish, intense, and somewhat acid. The acridity makes it disagreeable to take, and is not wholly done away with by combination with mucilage, syrups or glycerine. The dose of the fluid extract is from m xv to f. ʒ j.—*Ibid.*

**The Radical Treatment of Hernia.**—The conclusions were as follows: 1. Very favorable results have been obtained by the radical treatment of hernia; viz., ligature of the neck of the sac, extirpation of the sac, suture of the opening, with antiseptic precautions. The treatment is not dangerous, and has in most cases given good results. 2. Operation for hernia is indicated only in cases where the hernia cannot be kept *in situ* by a truss.—*Ibid.*

**Cubebs in Whooping-Cough.**—Dr. M. Sanchez has obtained rapid cures in cases of whooping-cough by the administration of an etherial tincture of cubebs in doses of four or five drops three times a day.—*Medical Times.*

